# SPANX-A/D protein subfamily plays a key role in nuclear organisation, metabolism and flagellar motility of human spermatozoa

**DOI:** 10.1038/s41598-020-62389-x

**Published:** 2020-03-27

**Authors:** Itziar Urizar-Arenaza, Nerea Osinalde, Vyacheslav Akimov, Michele Puglia, Iraia Muñoa-Hoyos, Marta Gianzo, Jose Antonio Rodriguez, Teresa Ganzabal, Blagoy Blagoev, Irina Kratchmarova, Nerea Subiran

**Affiliations:** 1 Department of Physiology. University of the Basque Country (UPV/EHU), Leioa, Bizkaia, Spain; 2 Biocruces Bizkaia Health Research Institute, Barakaldo, Bizkaia, Spain; 3 Department of Biochemistry and Molecular Biology, University of the Basque Country (UPV/EHU), Vitoria-Gasteiz, Araba, Spain; 4 Department of Biochemistry and Molecular Biology. University of Southern Denmark, Odense, Denmark; 5 Department of Genetics, Physical Anthropology and Animal Physiology, University of the Basque Country (UPV/EHU), Leioa, Spain; 6 Center for Reproductive Medicine and Infertility Quirón Bilbao, Bilbao, Spain

**Keywords:** Reproductive biology, Testis

## Abstract

Human sperm protein associated with the nucleus on the X chromosome (SPANX) genes encode a protein family (SPANX-A, -B, -C and -D), whose expression is limited to the testis and spermatozoa in normal tissues and to a wide variety of tumour cells. Present only in hominids, SPANX-A/D is exclusively expressed in post-meiotic spermatids and mature spermatozoa. However, the biological role of the protein family in human spermatozoa is largely unknown. Combining proteomics and molecular approaches, the present work describes the presence of all isoforms of SPANX-A/D in human spermatozoa and novel phosphorylation sites of this protein family. In addition, we identify 307 potential SPANX-A/D interactors related to nuclear envelop, chromatin organisation, metabolism and cilia movement. Specifically, SPANX-A/D interacts with fumarate hydratase and colocalises with both fumarate hydratase and Tektin 1 proteins, involved in meeting energy demands for sperm motility, and with nuclear pore complex nucleoporins. We provide insights into the molecular features of sperm physiology describing for the first time a multifunctional role of SPANX-A/D protein family in nuclear envelope, sperm movement and metabolism, considered key functions for human spermatozoa. SPANX-A/D family members, therefore, might be promising targets for sperm fertility management.

## Introduction

Currently, over 186 million people worldwide have infertility problems^1^, and male factors represent approximately 40–50% of clinical infertility cases^2^. Among other factors, anatomical or genetic abnormalities, problems in spermatogenesis or environmental factors^3^, are responsible for abnormal sperm parameters which contribute to male infertility. However, approximately 20–30% of men with normal sperm parameters have impaired fertility suggesting that male infertility can be caused by different deficiencies not yet described.

The sperm protein associated with the nucleus mapped to the X chromosome (SPANX) family is a testis-specific multigene family. SPANX genes encode proteins that belong to the so-called “cancer testis antigens” (CTA) family, a group of proteins whose expression is limited to the testis and spermatozoa in normal tissues and to a wide variety of tumours^4,5^. The SPANX family includes two subfamilies: the SPANX-N subfamily, composed of SPANX-N1, -N2, -N3, -N4 (in the locus Xq27.3) and -N5 (in the locus Xp11.22) that share a high level of sequence homology^6^; and the SPANX-A/D subfamily, which includes SPANX-A1, -A2, -C, -D (in the locus Xq27.2) and –B (in the locus Xq27.1) with high sequence homology^5,7^. Evolved from a common ancestor, the SPANX-N gene family is present in mice, rats and all primates, while the SPANX-A/D family is expressed only in hominids, including humans, bonobos and chimpanzees^8^. All SPANX proteins exhibit a similar post-meiotic expression pattern during spermatogenesis, appearing for the first time in haploid spermatids^4,7,9^. In mature spermatozoa, SPANX-N proteins are localised on the acrosome^6^, while SPANX-A/D proteins are expressed as dots in the cytoplasm and nucleus^4^. This difference in localisation could imply different roles for each subfamily in mature spermatozoa. Interestingly, the biological function of the SPANX-A/D family is still largely unknown.

Combining proteomics and molecular approaches, our aim was to elucidate the biological function of the SPANX-A/D subfamily in mature human spermatozoa. Our results describe for the first time a multifunctional role of SPANX-A/D in several key functions of human spermatozoa such as nuclear organization, metabolism and cilium movement.

## Results

### **Characterisation of SPANX-A/D protein expression, phosphorylation and localisation in human spermatozoa**

**Figure 1:**
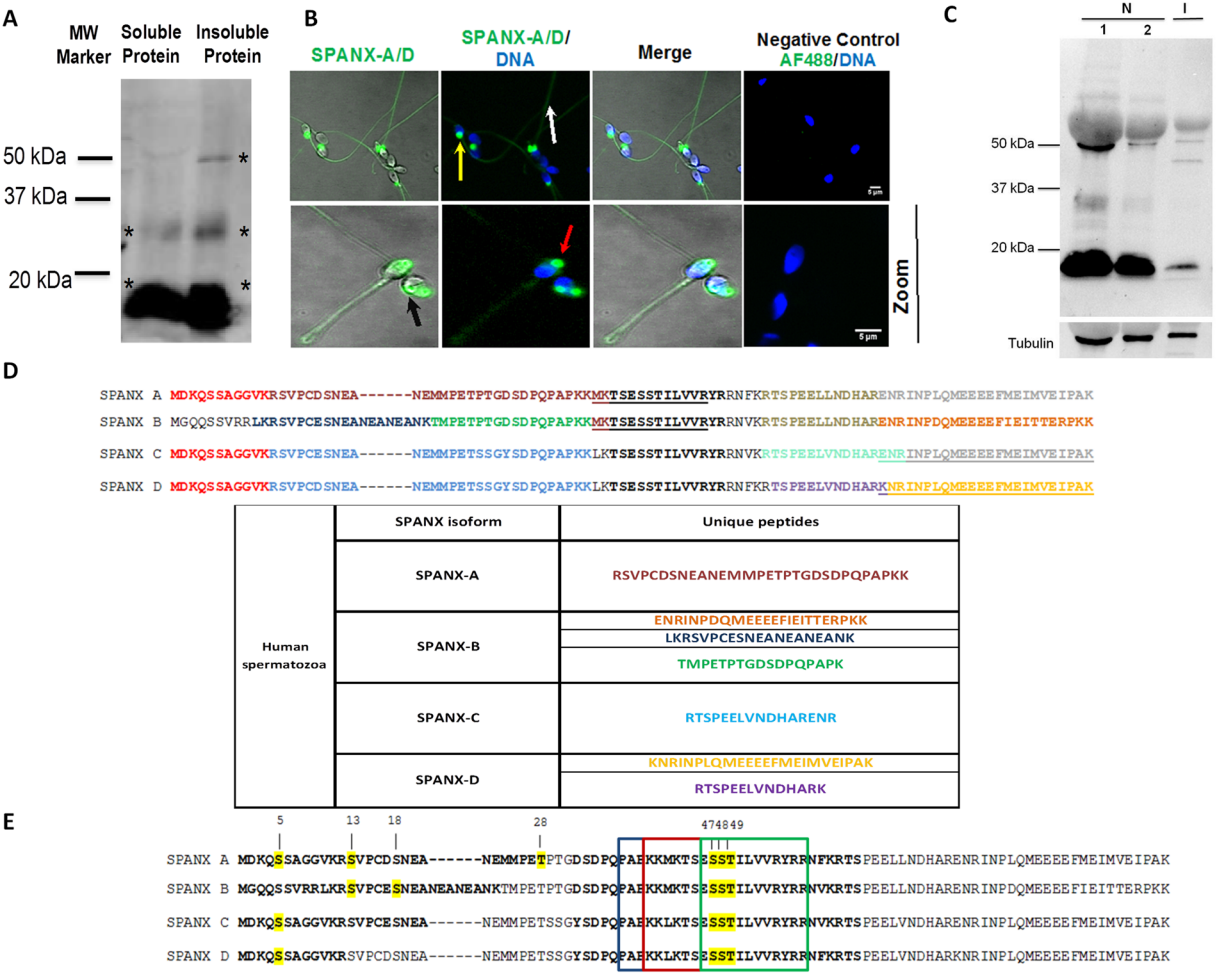
Characterisation of SPANX-A/D in human spermatozoa (**A**) Immunoblotting of SPANX-A/D protein in soluble and insoluble protein fractions (N = 5) **(B)** Immunofluorescence of SPANX-A/D protein in human spermatozoa. Negative controls were performed by omitting the primary antibody before secondary antiserum addition. Nuclei were stained with Hoechst and are represented in blue. Scale bar 5 μm (N = 4). (**C**) Immunoblotting of SPANX-A/D protein family in fertile (normozoospermic samples; F1: 750.000 cells; F2: 500.000 cells) and infertile (oligoteratoastenozoospermic sample; I:500.000 cells) patients. Tubulin was used as loading control. Representative image form 3 independent oligoteratoasthenozoospermic samples. (**D**) Table containing the unique peptides corresponding to each SPANX-A/D isoform found by LC-MS/MS in human spermatozoa. Comparison of each SPANX isoform sequence; each colour corresponds to the different peptides found by mass spectrometry. (**E**) Phosphorylation sites of each SPANX-A/D isoform found by LC-MS/MS are represented in yellow. The tree overlapping consensus NLSs are indicated in boxes.

The SPANX multigene family has been previously well characterised at the gene level^6,10^. To shed light on the role of the SPANX-A/D subfamily in human spermatozoa, we first carried out the subfamily characterisation at the protein level. Using immunoblotting, we evaluated the expression of endogenous SPANX-A/D in both soluble and insoluble protein fractions of human spermatozoa in 5 independent biological samples from normozoospermic patients. The anti-SPANX polyclonal antibody labelled two (37 kDa and 20 kDa) and three bands (50 kDa, 37 kDa and 20 kDa) in the soluble and insoluble fractions, respectively (Fig. 1A). No immunoreactivity was observed when the primary antibody was omitted (data not shown). We next determined the localisation of SPANX-A/D in human spermatozoa in normozoospermic samples (n = 4) by immunofluorescence. These studies confirmed the presence of SPANX-A/D proteins in human sperm cells (Fig. 1B). Strong immunoreactivity was observed at the neck of spermatozoa (60% of the SPANX-A/D-positive cells), on the acrosome (30% of the SPANX-A/D-positive cells) and in the nucleus (10% of the SPANX-A/D-positive cells), specifically in nuclear vacuoles (Fig. 1B). In addition, immunoreactivity was detected along the tail in 100% of the spermatozoa. No fluorescent staining was observed when the primary antibody was omitted.

In order to evaluate a potential role of SPANX-A/D in human sperm fertility, we measured its protein levels in normal (normozoospermia) and infertile men (oligoteratoasthenozoospermia), in which the concentration of sperm cells is reduced to less than 15 × 10^6^/ml, the sperm cell shape is abnormal and the sperm motility is diminished by over 40%. Western blot experiments performed with a small cohort of infertile patients (n = 3), showed a decreased SPANX-A/D protein levels in oligoteratoasthenozoospermic samples compared to normozoospermic semen (Fig. 1C).

Due to the high amino acid sequence homology among the members of the SPANX-A/D family^4^, we were not able to distinguish the different SPANX-A/D isoforms using commercially available polyclonal antibodies. Therefore, to circumvent this limitation, the presence of SPANX-A/D isoforms was evaluated by mass spectrometry (LC/MS-MS) in 10 normozoospermic samples and three technical replicates. In addition to common peptides shared by all isoforms, unique peptides belonging to SPANX -A, -B, -C and -D were detected by LC-MS/MS (Fig. 1D), proving the presence of all SPANX-A/D family members in humans. Of note, one of the identified common peptide (TSESSTILVVRYR) overlaps a putative nuclear localisation signal (NLS) that has been previously described for this protein subfamily^4,11^. Additionally, LC-MS/MS analyses were performed to search for phosphorylation sites on SPANX-A/D family members. Following the strict criteria described in Materials and Methods, we identified a total of 7 unique phosphorylation sites on SPANX-A/D family members (Fig. 1E). Specifically, seven phosphorylated residues were detected in SPANX-A (Ser5, Ser13, Ser18, Thr^28^, Ser^47^, Ser^48^ and Thr^49^), six in SPANX-B (Ser5, Ser13, Ser18, Ser^53^, Ser^54^ and Thr^55^) and four in SPANX-C/-D (Ser5, Ser^47^, Ser^48^ and Thr^49^). In addition, Ser^47^, Ser^48^, and Thr^49^ (corresponding to Ser^53^, Ser^54^ and Thr^55^ of SPANX-B) are located within the NLS of this protein family. Remarkably, we described for the first time those novel phosphorylation sites of SPANX-A/D protein subfamily members in human spermatozoa *in vivo*, since there are no previous evidences regarding these residues in PhosphoSitePlus database^12^.

### **Characterisation of novel SPANX-A/D phosphosites**

**Figure 2:**
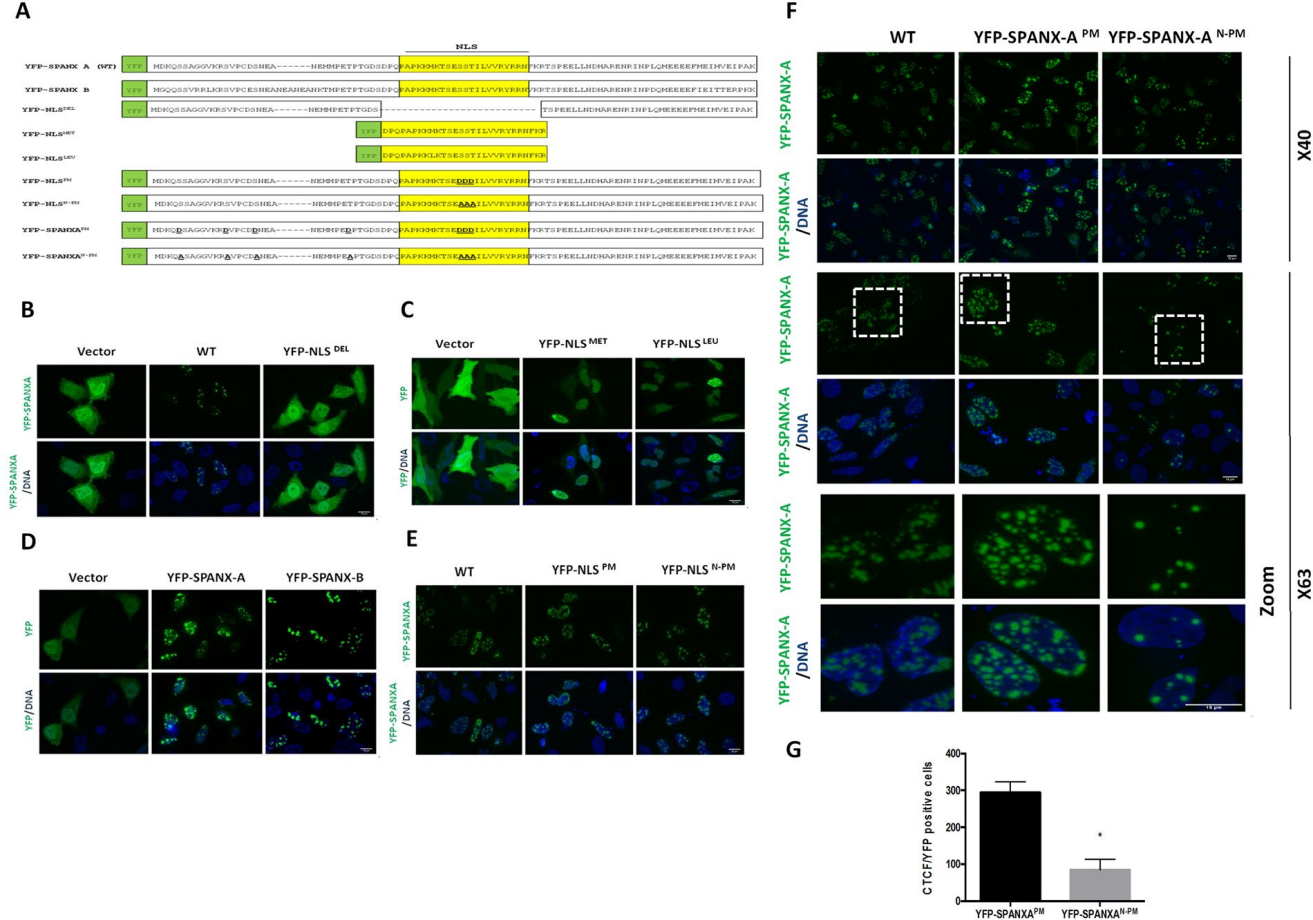
Mutagenesis studies of the SPANX-A/D protein subfamily in HeLa cells. (**A**) Schematic representation of all mutants transfected in HeLa cells. All expression plasmids were expressed with a YFP tail. YFP-SPANX-A was used as a wild-type sequence. The NLS appears drawn in yellow within the sequence of each YFP-mutant. Confocal microscopy images showing representative examples of HeLa cells transfected with expression plasmids encoding (**B**) YFP (vector), WT and WT mutant with deleted NLS (YFP-NLS^DEL^) (63×). DAPI was used to counterstain the nucleus (DNA panels) (N = 3); (**C**) YFP, the NLS of WT with methionine (YFP-NLS^MET^) and leucine (YFP-NLS^LEU^) at the 42nd position of the sequence (63×) (N = 3); (**D**) YFP, YFP-SPANX-A (WT) and YFP-SPANX-B (63×) (N = 3). Confocal images of phosphomimetic (YFP-NLS^PM^) and dephosphomimetic mutants (YFP-NLS^N-PM^) of NLS at the 47th, 48th and 49th positions (63×) (N = 3); (**F**) Confocal images of phosphomimetic (YFP-SPANX-A^PM^) and dephosphomimetic mutants (YFP-SPANX-A^N-PM^) of the SPANX-A, modifying all identified phosphosites at the 5th, 13th, 18th, 28th, 47th, 48th and 49th positions of the protein sequence (40x and 63×). The zoomed section appears framed by a dotted line. DAPI was used to stain the nuclei (DNA panels). Scale bar: 15 μm. (N = 3) (**G**) Graph showing the corrected total cell fluorescence (CTCF) intensity/YFP positive cells of the YFP-SPANX-A^PM^ vs YFP-SPANX-A^N-PM^ mutants. The data shown in the graph correspond to the mean of three independent experiments, and the error bars indicate the SEM. *P < 0.05 (Student’s t-test).

Considering that several SPANX-A/D phosphosites were located on a putative NLS, we evaluated their role on nuclear translocation. We used phosphomimetic approaches and transfection of YFP-tagged proteins to determine whether the phosphorylation state of SPANX-A/D influenced the nuclear translocation of SPANX-A/D (Fig. 2A). Due to the impossibility of performing transfections in human spermatozoa, we performed these experiments in HeLa and HEK293T cell lines. First, we evaluated the functionality of the putative NLS using NLS-null SPANX mutants (YFP-NLS^DEL^) (Fig. 2B and Supplementary Fig. 1A). YFP-SPANX-A exhibited prominent nuclear localisation, accumulating in nuclear dots and in the periphery of the nucleus. In contrast, the YFP-NLS^DEL^ mutant was diffusely localised throughout the cell (Fig. 2B and Supplementary Fig. 1A), indicating that the NLS is required for the nuclear accumulation of SPANX-A. Of note, the NLS sequences of SPANX-A/-B and SPANX-C/-D differ at a single amino acid position. To evaluate whether this specific amino acid difference affects NLS functionality, we designed two different constructs encoding the NLS with either a leucine (YFP-NLS^LEU^) or a methionine (YFP-NLS^MET^) residue (Fig. 2A). Both constructs led to similar nuclear localisation of the YFP-tagged proteins (Fig. 2C and Supplementary Fig. 1B), suggesting that the leucine/methionine sequence variation does not significantly affect the NLS functionality. Then, to evaluate whether the difference in length between SPANX-A and SPANX-B, which is 6 amino acids longer than SPANX-A, correlates with a different subcellular distribution; we designed a construct encoding YFP-SPANX-B. Both YFP-SPANX-A and YFP-SPANX-B exhibited similar nuclear localisation in both cell lines (Fig. 2D and Supplementary Fig. 1C). In fact, a 3D reconstruction (Supplementary Fig. 2) revealed that both constructs were organised in nuclear dots of variable size showing the presence of larger dots localised inside the nucleus and smaller dots with epinuclear localisation along the nuclear envelope. Finally, phosphomimetic approaches were used to replace the three phosphosites inside the NLS (Ser^47^, Ser^48^ and Thr^49^) with aspartic acid (D) or alanine (A), mimicking the phosphorylated (YFP-NLS^PM^) and non-phosphorylated (YFP-NLS^N-PM^) states of the NLS, respectively. We detected no significant difference in the localisation of the wild-type (WT), YFP-NLS^PM^ and YFP-NLS^N-PM^ constructs (Fig. 2E and Supplementary Fig. 1D). Therefore, we next replaced all the phosphosites identified in SPANX-A/D (Ser^5^, Ser^13^, Ser^18^, Thr^28^, Ser^47^, Ser^48^ and Thr^49^) with aspartic acid or alanine to mimic the phosphorylated (YFP-SPANX-A^PM^) and non-phosphorylated (YFP-SPANX-A^N-PM^) versions of the protein (Fig. 2A), respectively. Both constructs were able to translocate into the nucleus of HeLa (Fig. 2F) and HEK293T (Supplementary Fig. 1E) cells and accumulated in nuclear dots, indicating that the phosphorylation state is not a crucial determinant of the nuclear translocation of the protein. Nevertheless, the phosphomimetic YFP-SPANX-A^PM^ mutant showed larger and more intense dots inside the nuclei of HeLa and HEK293T cells than WT or non-phosphorylated mutant (Fig. 2F,G and Supplementary Fig. 1E,F), suggesting that the phosphorylation state of the protein might influence SPANX-A/D protein complex stabilisation.

### **Functional characterisation of the SPANX-A/D protein family in human spermatozoa**

**Figure 3:**
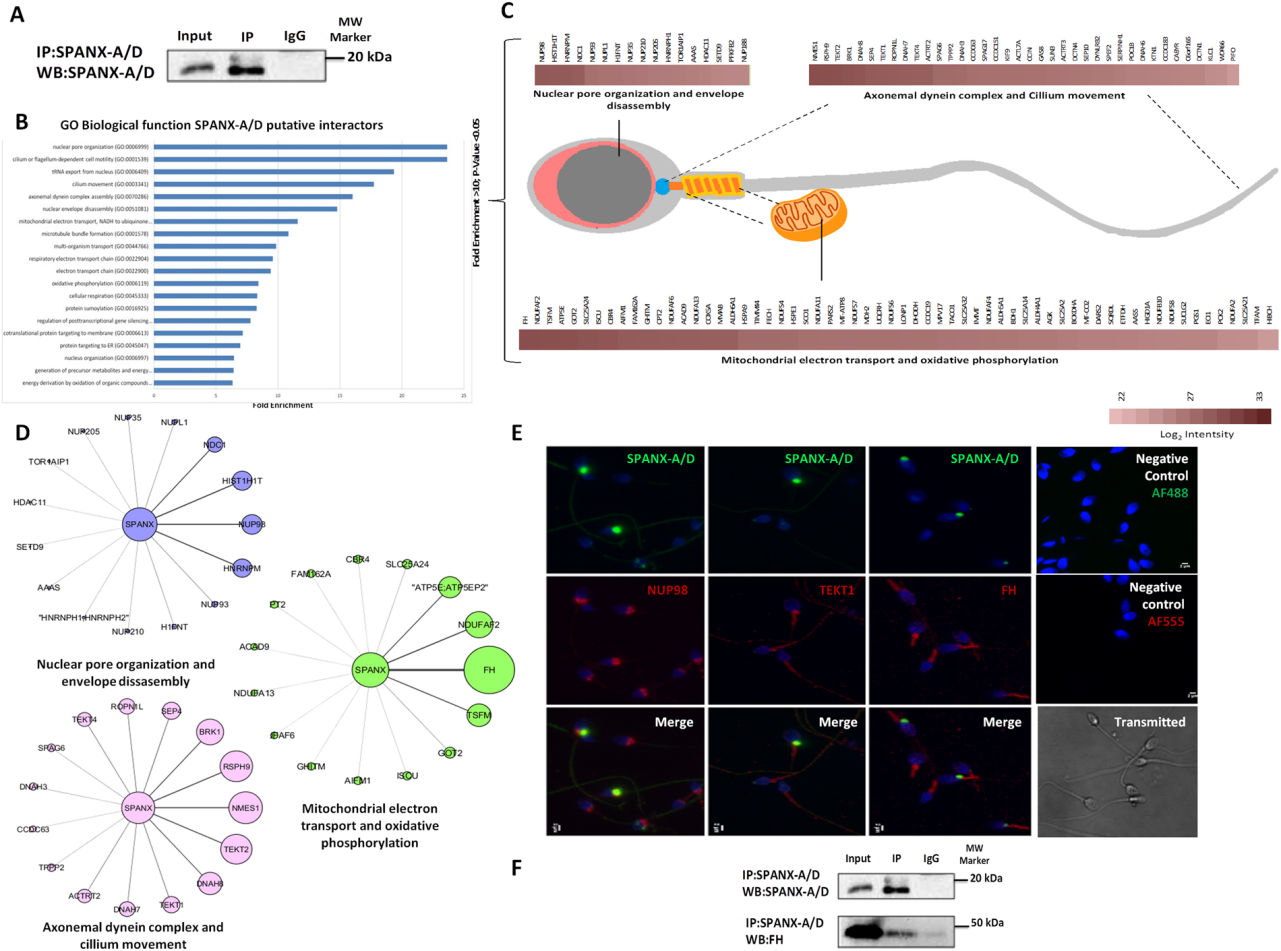
Study of the SPANX-A/D interactome in human spermatozoa. (**A**) Immunoprecipitation of SPANX-A/D in human spermatozoa. (N = 3) (**B**) GO analysis based on the biological functions of co-precipitated proteins together with SPANX-A/D in human spermatozoa. The twenty most enriched processes are shown. P < 0.05. (**C**) Representative scheme of principal interactors of SPANX-A/D based on their localisation in human spermatozoa and biological function. Only the 45 most intense interactors with no enriched biological function are shown. (**D**) Schematic representation of the 15 most intense interactors of each biological process. The size of the nodes and the thickness of the edges represent the intensity of each potential interactor. (**E**) Co-localisation assays by immunocytochemistry of SPANX-A/D (green) and NUP98, TEKT1 and FH (red). For the specificity of the secondary antisera, the primary antibodies were omitted. The nuclei were stained with Hoechst and are represented in blue. The transmitted image of human sperm is included to show the integrity of the sample. Scale bar: 2 μm. (N = 3) (**F**) Co-immunoprecipitation of SPANX-A/D and FH. Negative controls were performed with non-specific IgG antibodies for immunoprecipitation.

To uncover the physiological role of the SPANX-A/D subfamily in human normozoospermic spermatozoa, we studied the interactome using a label-free quantitative proteomics approach. Considering that nearly 30% of the proteins expressed in human spermatozoa are cell-specific and not found in any other cell types, we searched for SPANX-A/D interactors in human spermatozoa using the endogenous SPANX-A/D protein family as bait (Fig. 3A and Supplementary Fig. 3). SPANX-A/D proteins from a pool of 80 normozoospermic samples were immunoprecipitated with the polyclonal commercial anti-SPANX antibody (Abcam, ab119280) and co-precipitating proteins were digested in solution with trypsin to be analysed by LC-MS/MS in 4 technical replicates. A non-specific rabbit IgG that does not bind SPANX-A/D was used as a negative control in parallel pull-down assays. A total of 307 potential SPANX-A/D interactors were identified. We considered as potential SPANX-A/D interactors those proteins that were confidently detected in the four SPANX-A/D pulldowns but in not in the negative control pull-down assays (Supplementary Table S1). Gene ontology (GO) analysis (Fig. 3B) revealed that potential SPANX-A/D interactors are mainly involved in nuclear pore organisation (23.65-fold enrichment), cilium or flagellum-dependent motility (23.65-fold enrichment), assembly of the axonemal dynein complex (16.02-fold enrichment) and mitochondrial electron transport (11.58-fold enrichment). These results suggest that the SPANX-A/D protein subfamily may exert a multifunctional role in human spermatozoa. A more in-depth analysis (Fig. 3C,D) revealed that several nuclear proteins, such as testis-specific H1 histone (H1FNT) or histone 1 (HISTH1T); chromatin regulators, such as SET domain-containing protein 9 (SETD9) or histone deacetylase 11 (HADC11); and nucleoporins (NUPs), such as NUP98, NUP93, NUP35 or NUP188, co-precipitated with SPANX-A/D. Specifically, NUP98 co-localised with SPANX-A/D in the neck of the spermatozoon (Fig. 3E). On the other hand, the structural component of cilia and flagellar microtubules, such as dyneins and tektins, also co-precipitated with SPANX-A/D. TEKT1 co-localised with the protein family in the neck of the spermatozoa and along the tail (Fig. 3E). Finally, proteins related to mitochondrial metabolic processes, such as fumarate hydratase (FH) that showed the highest MS intensity, NADH dehydrogenase alpha subcomplex assembly factor (NDUFAF2), and mitochondrial ATP synthase subunit epsilon (ATP5E) (Fig. 3C,D) were also identified as potential SPANX-A/D interactors. Specifically, FH co-localised with SPANX-A/D in the neck of the spermatozoon (Fig. 3E) and physical interaction between both proteins FH and SPANX-A/D in human spermatozoa was confirmed by co-immunoprecipitation assays (Fig. 3F and Supplementary Fig. 3).

## Discussion

**Figure 4:**
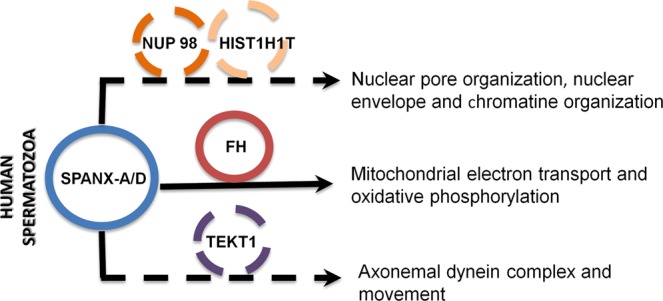
Role of SPANX-A/D in human spermatozoa. Schematic representation of molecular mechanisms in which SPANX-A/D is involved in human spermatozoa. Physical interactions are represented with a continuous line, while the co-expression is shown with a discontinuous line.

SPANX-A/D is a multigene family mapped on the X chromosome exhibiting testis-specific expression and mRNA/protein localisation exclusively in post-meiotic spermatids^5,11^. The presence of the SPANX-A/D subfamily has been widely studied in a broad variety of cancer types over the last twenty years^4,10,13^. In 2001, these proteins were described for the first time in testis and spermatozoa^4^; however, the biological role in sperm fertility is largely unknown. Through a combination of proteomic analysis, phosphomimetic assays and molecular approaches, our results indicate that SPANX-A/D is a multifunctional protein family that may play a key role in human sperm physiology (Fig. 4). In line with other studies^4,9,11^, we confirmed the presence of all isoforms of the SPANX-A/D subfamily protein, which are localised in the nucleus, cytoplasm and in the flagellum of human spermatozoa. Specifically, SPANX-A/D is located at the nuclear vacuoles of human spermatozoa, which are known to be related to sperm genome instability and, therefore, to sperm fertility^14^. In fact, although further studies are needed to confirm this fact in a larger cohort of patients, our results suggest that SPANX-A/D protein levels are lower in infertile men with oligoteratoasthenozoospermia than in fertile men. This is consistent with the fact that asthenozoospermic semen samples present a lower expression of SPANX-B at protein levels than normozoospermic samples^15^, suggesting that the presence of SPANX-A/D in mature spermatozoa may be crucial for the maintenance of human sperm functions.

On the other hand, we describes for the first time novel SPANX-A/D phosphorylated residues in normal human spermatozoa *in vivo*, in spite of some phosphosites have been previously reported in different cancer cell types (PhosphoSitePlus database 11076738, 11076741, 9161501, 50832232, 11076732, and 11076735). Proteomic approaches reveal the presence of seven novel phosphosites along the SPANX-A/D protein sequences (Ser5, Ser^13^, Ser^18^, Thr^28^, Ser^47^, Ser^48^, and Thr^49^) and, interestingly, three of them lie within the NLS. In order to evaluate its potential role in cytoplasm-nuclear transport, we carried out phosphomimetic analyses. The phosphorylation state of neither the NLS nor the whole protein is determinant of SPANX-A nuclear translocation, but this post-translational modification may be important for promoting the stabilisation of the SPANX-A dots into nucleoplasm.

Taking into account the widespread localisation of SPANX-A/D, this protein family might play different roles in human spermatozoa. Since the expression of the SPANX-A/D subfamily appears to be restricted to hominids^7^, knockout mice are not useful models for elucidating its biological function. Importantly, human spermatozoa present a unique proteome, and at least 30% of this proteome is made up of sperm-specific proteins^16^. Therefore, in an attempt to clarify the physiological role of the protein family, we carried out an interactome analysis of endogenous SPANX-A/D protein family in human spermatozoa from normozoospermic samples, using it as bait in immunoprecipitation assays. Our results indicate that SPANX-A/D protein family plays a multifunctional role in human spermatozoa. We have identified 307 proteins related to nuclear envelope and chromatin organisation, mitochondrial metabolism and flagellum-dependent motility that co-immunoprecipitate with SPANX-A/D. According to the interactome analysis, SPANX-A/D co-precipitates with several components of the nuclear pore complex such as NUP98, NUP93, NUP35 and NUP188. Our results, together with the fact that previous studies localise SPANX-A/D in nuclear envelope organisation^4^, indicate that SPANX-A/D might be part of nuclear pore envelope. SPANX-A/D and NUP98 co-localise in the neck of spermatozoa, which is considered a redundant nuclear envelope in mature spermatozoa^17^. Besides nucleocytoplasmic transport regulation, nuclear pore complexes directly interact with chromatin to regulate transcription^17^. Interactome approach also identifies proteins involved in chromatin regulation as potential SPANX-A/D interactors such as several histones (testis-specific H1 histone (H1FNT) and histone 1 (HISTH1T)) and chromatin regulators (SET domain-containing protein 9 (SETD9) and histone deacetylase 11 (HADC11)). This is consistent with the fact that SPANX-A/D protein family is present inside nuclear vacuoles, which have been widely associated with chromatin condensation failure and genomic instability^18–20^,. Therefore, our results indicate that SPANX-A/D might play an important role in sperm physiology by regulating chromatin organisation in mature spermatozoa.

Additionally, SPANX-A/D subfamily also co-precipitates with proteins involved in other physiological functions related to mitochondrial metabolism such as FH, cilium organisation and flagellum-dependent motility such as tektins and dyneins. The contribution of the metabolic/energy pathways in sperm motility has been previously reported since the beat frequency of the flagellum is directly related to the production rate of energy from ATP^21–24^. The axonemal dyneins generate force through the hydrolysis of the ATP created in the mitochondria and bind to the structural components of the mictubules, such as tektins, to actively participate in flagellar movement^25^. In line with this fact, the expression of FH and TEKT1 is lower in semen samples with poor sperm motility (asthenozoospermia)^26^, indicating that SPANX-A/D family, through the interaction with both proteins FH and TEKT1, could be essential in human spermatozoa to meet energy demands for motility.

Overall, a more detailed knowledge of the biological role of the SPANX-A/D protein family is indispensable for understanding human sperm fertility and may be useful for elucidating the aetiology of many cases of male infertility. Although further studies need to be performed in order to evaluate the role of each isoform in human spermatozoa, SPANX-A/D protein family may be a promising target for sperm fertility management in humans as it plays a multifunctional role in nuclear envelope, sperm movement and metabolism.

## Methods

### Samples and isolation of spermatozoa

Ethical approval for this study was obtained from the Ethics Committee of the University of the Basque Country (CEISH-UPV/EHU (M10/2016/254)), and all experiments were performed in accordance with relevant guidelines and regulations. Informed consent was also obtained from all participants. Freshly ejaculated semen was collected from 112 men undergoing routine semen analysis at the Center for Reproductive Medicine and Infertility Quirón (Bilbao, Spain). The men had normal sperm parameters (normozoospermia) according to World Health Organization standards^27^. The infertile spermatozoa corresponded to 3 different oligoteratoasthenozoospermic samples (the concentration of sperm cells is reduced to less than 15 × 10^6^/ml, their motility is diminished by over 40% and have abnormal sperm shape^27^). Semen samples were obtained by masturbation after 3–4 days of sexual abstinence and processed immediately upon liquefaction (at 37 °C for 30 min). Spermatozoa were capacitated by the *swim-up* procedure and resuspended in G-IVF (Vitrolife, Goteborg, Sweden) supplemented with 1% bovine serum albumin for 3 h at 37 °C under 5% CO_2_.

### Plasmids, cloning procedures and mutagenesis

Locus position of SPANX-A/D is described by Kouprina *et al*.^7^. To generate the plasmids encoding YFP–SPANX-A (WT), YFP-SPANX-B, YFP-SPANX-A-NLS Deleted (YFP-NLS^DEL^), YFP-NLS^MET^, YFP-NLS^LEU^, YFP-NLS^PM^, YFP-NLS^N-PM^, YFP-SPANX-A^PM^ and YFP- SPANX-A^N-PM^, each cDNA was synthetised as HindIII/BamHI fragments (gBlocks Gene Fragments, Integrated DNA Technologies, CA, USA) and subcloned into pEYFP-C1 plasmids (Clontech, Mountain View, CA, USA). All of the constructs generated were subjected to DNA sequencing (Stabvida, Caparica, Portugal), and the absence of any unwanted mutation was confirmed. The sequences of the oligonucleotides used in cloning and mutagenesis are available upon request. The amino acid sequences coding each YFP protein are detailed below in Fig. 2A.

### Transfections and confocal microscopy

HeLa cells (human epithelial cervical cancer cells) and HEK293T cells (human epidermal kidney cells) were cultured in Dulbecco’s modified Eagle’s medium supplemented with 10% FBS, 2 mM L-Glutamine, 100 U/mL penicillin, and 100 μg/mL streptomycin (all from Invitrogen, Carlsbad, CA, USA). Twenty-four hours before transfection, the cells were seeded onto 12-well or 6-well tissue culture plates. HEK293T and HeLa cell line plasmid transfections were carried out with X-tremeGENE 9 transfection reagent (Roche Diagnostics, Indianapolis, IN, USA) according to the manufacturer’s protocol. To evaluate the transfection efficiency, cells growing on sterile coverslips were fixed with 3.7% formaldehyde in PBS for 30 min and then assembled onto slides using Vectashield Antifade Mounting Medium with DAPI (Vector Laboratories, USA). To avoid bias in the quantification of YFP fluorescence, slides were encoded, and images were taken and examined unaware of the identity of the samples. Image analysis with ImageJ software was used to quantify the intensity of YFP fluorescence.

### Western blotting

For protein expression analyses, isolated sperm cells were lysed using ice-cold RIPA buffer (50 mM Tris-HCl, pH 7.5, 150 mM NaCl, 1% NP-40, 1 mM EDTA, 0.25% sodium deoxycholate, 1 mM sodium pervanadate, 5 mM beta-glycerophosphate, 5 mM NaF, complete protease inhibitor cocktail (Complete tablets, Roche)). After protein homogenisation and sonication (20% amplitude, 10 pulses, three times, Labsonic B.Braun International), proteins were separated both in soluble and insoluble fractions by centrifugation at 13000 g, 4 °C, 15 min. The protein concentration was measured by the Bicinchoninic Acid (BCA) assay method.

For the characterisation on SPANX-A/D protein family in human spermatozoa, thirty micrograms of each insoluble and soluble protein fraction were prepared. For the comparison of SPANX-A/D protein levels in normozoospermic and infertile spermatozoa, fifty and thirty micrograms of insoluble protein fraction were prepared. Protein extracts were diluted in Laemmli sample buffer containing dithiothreitol (DTT) and were boiled for 5 min. All samples were loaded onto 12% resolving gels and separated by one-dimensional sodium dodecyl sulfate-polyacrylamide gel electrophoresis (SDS-PAGE).

Proteins were then transferred to polyvinylidene fluoride membranes using the Mini Trans-Blot Electrophoretic Transfer System (Bio-Rad Laboratories, Hercules, CA, USA). After transfer, the membranes were blocked with Blotto (20 mM Tris-HCl, pH 7.5, 0.15 M NaCl, and 1% Triton X- 100) containing 5% bovine serum albumin (BSA) (Sigma-Aldrich) for 1 h at room temperature and then incubated with a polyclonal rabbit anti-SPANX antibody (1:500) diluted in 5% BSA (ab119280, Abcam), a monoclonal mouse anti-tubulin antibody (1:10000) (T9026, Sigma-Aldrich) and a monoclonal mouse anti-FH antibody (sc-393992, Santa Cruz Biotechnology) (1:200). After washing in Blotto buffer, the membrane was incubated for 1 h with a peroxidase-conjugated goat anti-rabbit IgG antibody (1:1000) (goat anti-rabbit IgG-HRP, sc-2004, Santa Cruz Biotechnology) or a peroxidase-conjugated donkey anti-mouse IgG antibody (1:2000) (donkey anti-mouse IgG-HRP, sc-2314, Santa Cruz Biotechnology).

### Protein in-gel digestion

For the study of SPANX-A/D isoforms in human spermatozoa, the soluble and insoluble protein fractions were loaded onto a precast gradient NuPAGE 4–12% Bis-Tris Protein gel (Invitrogen) for further visualisation with Colloidal Blue (Invitrogen). Whole gel lines were cut into slices and subjected to reduction with 10 mM dithiothreitol (DTT), alkylation with 55 mM chloroacetamide (CAA) and protein digestion by incubating with trypsin overnight at 37 °C. The resulting tryptic peptides were extracted from the gel by serial incubations with 100% acetonitrile (ACN) and 30% ACN/3% trifluoroacetic acid (TFA). Finally, the solutions obtained in all the incubations were dried down in a vacuum centrifuge. Peptides derived from slices from each lane were concentrated and desalted using C18 stage tips (made in house using Empore Disc-C18 Agilent Life Science) for further analysis by LC-MS/MS.

### Immunoprecipitation and in-solution digestion

For the SPANX-A/D interactome in human spermatozoa, cells were lysed using commercial Co-IP buffer (Thermo Scientific) with complete protease and phosphatase inhibitor cocktail (Roche) (quadruplicate). After protein homogenisation and sonication (20% amplitude, 10 pulses, three times), proteins were separated in soluble fractions by centrifugation at 13000 g and 4 °C for 15 min. The soluble protein fraction was incubated for 4 h at 4 °C with magnetic beads (PureProteome Protein A Magnetic Beads, Millipore) conjugated to an anti-SPANX antibody (ab119280, Abcam). This polyclonal commercial anti-SPANX antibody recognises an amino acid sequence (GDSDPQPAPKKMKTSE) corresponding to all SPANX-A, -B, -C and -D isoforms: As a negative control, rabbit IgGs (X0903, DAKO) were used at the same concentration as the antibody. Immune complexes were independently recovered and washed. Elution of the immunocomplexes was carried out with 8 M guanidinium hydrochloride at pH 8 and 70 °C for 15 min. The proteins were then reduced and alkylated, followed by in-solution digestion with LysC/trypsin. Peptides derived from each sample were concentrated and desalted using C18 stage tips (made in house using Empore Disc-C18 Agilent Life Science) for analysis by LC-MS/MS.

### LC-MS/MS

Acidified peptide mixtures were separated by online C18 reverse-phase nanoscale liquid chromatography and analysed by tandem mass spectrometry (LC-MS/MS). MS analysis was performed on a Q-Exactive HF mass spectrometer (Thermo Scientific, Bremen, Germany) connected to an EASY-nanoLC 1000 System (Thermo) using a nanoelectrospray ion source (Proxeon Biosystems, Odense, DK). Survey full-scan MS spectra (m/z range, 200–2000; resolution 60,000 at m/z 400) were acquired in the Orbitrap followed by the fragmentation of the twelve most intense multiply charged ions. Ions selected for MS/MS were placed on a dynamic exclusion list for 45 s. To improve mass accuracy, internal real-time lock mass calibration was enabled. Additional mass spectrometric parameters included a spray voltage of 2.3 kV, no sheath and auxiliary gas flow, and the temperature of the heated capillary was 275 °C. All raw files were searched against the combined human database 2015.08 UniProt (with 42,122 sequence entries) and TrEMBL (with 49,496 sequence entries) using MaxQuant platform versions 1.5.2.8 and 1.5.3.30 with an Andromeda search engine. Precursor and fragment tolerances were 4.5 and 20 ppm, respectively. A peak list was generated using the Quant element of MaxQuant using the following parameters: a maximum of 2 missed cleavages were allowed, and enzyme specificity was set to trypsin. In addition, carbamidomethyl (C) was chosen as a fixed modification, and variable modifications included oxidation (M), deamidation (NQ) and Phospho_STY (STY). The peptide and protein FDR 0.01; site FDR 0.01; max. peptide PEP, 1; min. peptide length, 7; and min. unique peptides and peptides, 1. For protein quantitation, only unmodified peptides and peptides modified by acetyl (protein N-terminus), oxidation (Met) and deamidation (NQ) were used. According to the protein group assignment performed by MaxQuant, the identified proteins were determined after removing the contaminants, reverse hits and proteins identified only by site. Moreover, we took into account those proteins with ≥2 identified peptides and ≥1 unique peptides. On the other hand, the phosphopeptide data were filtered by FDR < 1%, and only the phosphosites displaying a localisation probability above 0.75 were considered confident phosphorylated sites (Class I sites). Finally, the output data were analysed in the Perseus 1.6.0.7 bioinformatics analysis program.

### Bioinformatics analysis

Perseus software (v.1.6.0.7) was employed for the calculation of the statistical significance between the SPANX pull-down samples and the negative control in human spermatozoa. In the case of human spermatozoa, we filtered the protein list assuming as putative interactors those proteins identified with at least two peptides and containing a minimum of three unique peptides. The study was performed in quadruplicate. Moreover, we accepted only those proteins that appeared in all four replicas of the SPANX-A/D immunoprecipitation samples but not in any of the negative controls as candidate proteins. The PANTHER (v13.1) functional annotation tool (http://geneontology.org/) was used to detect the overrepresented gene ontology (GO) term “biological process” within the possible SPANX interactors in human spermatozoa. The Cytoscape computational tool (v.3.5.1) was used for the visualisation of the SPANX-A/D interactome in human spermatozoa.

### Indirect immunofluorescence and confocal microscopy

Isolated human sperm cells were fixed in 4% paraformaldehyde for 10 min, permeabilised in 0.5% Triton X-100 for 10 min and blocked for 30 min with 10% (v/v) foetal bovine serum (FBS) in PBS. For HEK293T and HeLa cell lines, however, permeabilisation was performed using 0.2% Triton X- 100 for 10 min and blocking for 30 min with 10% (v/v) FBS in PBS. Then, the samples were incubated overnight at 4 °C with polyclonal rabbit anti-SPANX (ab119280, Abcam) (1:500), monoclonal mouse anti-NUP98 (sc-74578, Santa Cruz Biotechnology) (1:50), monoclonal mouse anti-TEKT1 (sc-398507, Santa Cruz Biotechnology) (1:50) and monoclonal mouse anti-FH (sc- 393992, Santa Cruz Biotechnology) (1:50) antibodies. Secondary antibody incubations included Alexa Fluor 488 donkey anti-rabbit IgG (1:2000) (Molecular Probes, Oregon USA), Alexa Fluor 555 goat anti-mouse IgG (1:1000) (Thermo Scientific) and Alexa Fluor 663 goat anti-rabbit IgG (1:1000) (Thermo Scientific) antibodies. Controls for the specificity of the secondary antisera were simultaneously performed by omitting the primary antiserum before addition of the secondary antiserum. Nuclei were stained with Hoechst 33258 at 10 µg/mL, and slides were assembled with Fluoromount G (Molecular Probes). Finally, the samples were examined using confocal microscopy (Zeiss Apotome 2, Jena, Germany) at the High-Resolution Microscopy Facility (SGIKER UPV/EHU). Image analysis was conducted using ImageJ software.

## Supplementary information

Supplementaryinformation
