# Supplementary material for: SPANX-A/D protein subfamily plays a key role in nuclear organisation, metabolism and flagellar motility of human spermatozoa
Source: Sci Rep. 2020 Mar 27;10:5625. doi: 10.1038/s41598-020-62389-x (PMC7101357; doi:10.1038/s41598-020-62389-x)
Supplement: Supplementary file 1 — Supplementaryinformation [file 41598_2020_62389_MOESM1_ESM.doc]

**SPANX-A/D protein subfamily plays a key role in nuclear organisation, metabolism and flagellar motility of human spermatozoa**

Itziar Urizar-Arenaza^1^**^,2^**, Nerea Osinalde-Moraleja^3^, Vyacheslav Akimov^4^, Michele Puglia^4^, Iraia Muñoa-Hoyos^1,2^, Marta Gianzo^1^, Jose Antonio Rodriguez^5^, Teresa Ganzabal^6^, Blagoy Blagoev^4^, Irina Kratchmarova^4,#^* and Nerea Subiran^1,2,#^*.

1 Department of Physiology. University of the Basque Country (UPV/EHU)

2 Biocruces Bizkaia Health Research Institute. Bizkaia. Spain

3 Department of Biochemistry and Molecular Biology. University of the Basque Country (UPV/EHU), Vitoria-Gasteiz, Spain

4 Department of Biochemistry and Molecular Biology. University of Southern Denmark. Odense. Denmark

5 Department of Genetics, Physical Anthropology and Animal Physiology, University of the Basque Country (UPV/EHU), Leioa, Spain

6 Center for Reproductive Medicine and Infertility Quirón Bilbao, Bilbao, Spain

^#^ These authors contributed equally to the work.

*To whom correspondence should be addressed:

- Nerea Subiran Ciudad, Department of Physiology. Faculty of Medicine and Nursing. University of Basque Country. 48940. Leioa, Bizkaia, Spain. +34 946015673. [nerea.subiran@ehu.eus](mailto:nerea.subiran@ehu.eus)
- Irina Kratchmarova, Department of Biochemistry and Molecular Biology. University of Southern Denmark. 5320, Odense, Denmark. +45 65502494. [ihk@bmb.sdu.dk](mailto:ihk@bmb.sdu.dk)

# Supplementary Figures

# Supplementary Figure 1

**Vector**

**A**

**YFP-SPANX-A**


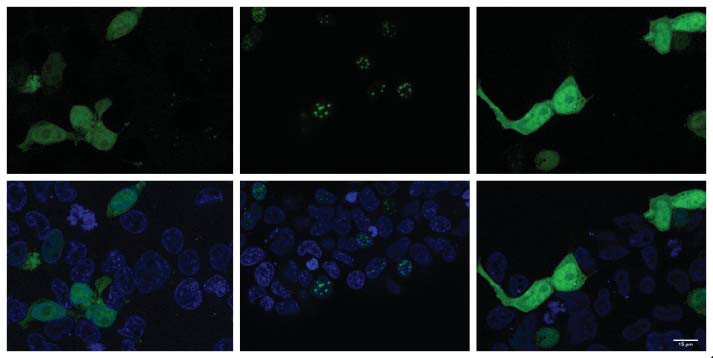


**WT YFP-NLS^DEL^**

**Vector**

**B**

**YFP-SPANXA**

**/DNA**

**YFP**

**YFP-NLS MET**

**YFP-NLS LEU**


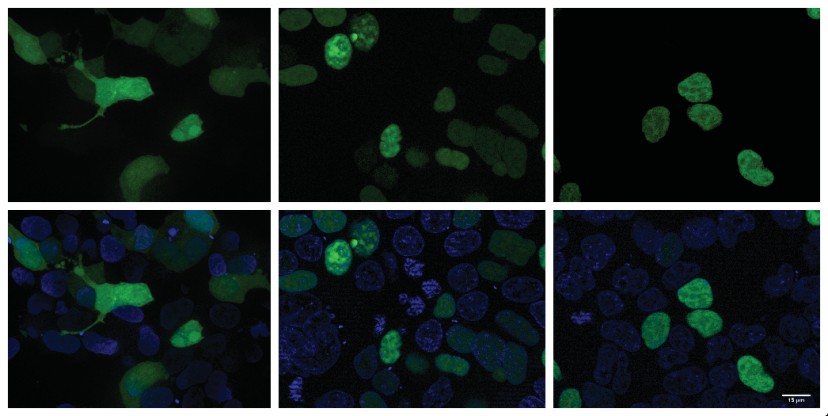


**Vector YFP-SPANX-A YFP-SPANX-B**

**YFP/DNA**

**C**

**
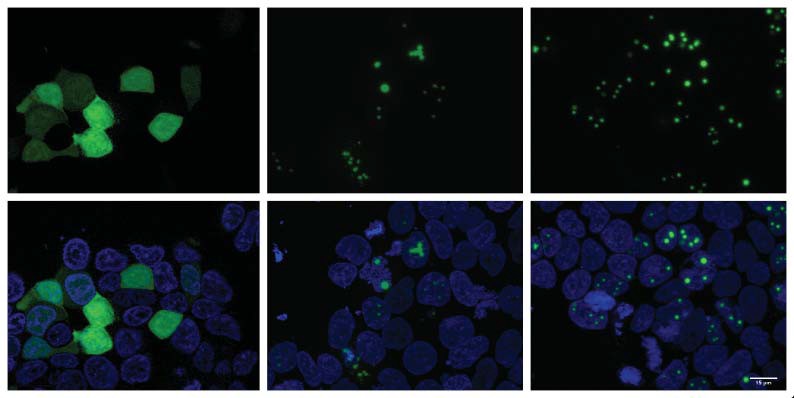
**

**YFP**

**YFP/DNA**

**YFP-SPANX-A**

**/DNA**

**YFP-SPANX-A**

**/DNA**

**YFP-SPANX-A**

**YFP-SPANX-A**

**/DNA**

**YFP-SPANX-A**

**YFP-SPANX-A**

**/DNA**

**YFP-SPANX-A**

**D**

**WT**

**E**

**WT**

**YFP-SPANX-A**

**YFP-SPANX-A PM**

**YFP-NLSPM**

**YFP-NLSN-PM**

**Zoom**

**YFP-SPANX-A N-PM**

**X40**

**X63**

**150**

**F**

**100**

**CTCF/YFP positive cells**

**50** *

**0**

**YFP-SPANX-APM YFP-SPANX-AN-PM**

**Supplementary Figure 1. Mutagenesis studies of SPANX-A/D protein subfamily in HEK293T cells.**

Confocal microscopy images showing representative examples of HEK293T cells transfected with expression plasmids enconding **(A)** YFP (vector), WT and WT mutant with the deleted NLS (YFP-NLS^DEL^) (x63). DAPI was used to counterstain the nucleus (DNA panels) (N=3) **(B)** YFP, the NLS of the WT with Methionine (YFP-NLSMET) and Leucine (YFP-NLSLEU) at the 42nd positions of the sequence (x63). (N=3) **(D)** YFP, YFP-SPANX-A (WT) and YFP-SPANX-B (x63). (N=3) **(e)** WT and the phospho-mimetic (YFP-NLSPM) and dephospho-mimetic mutants (YFP-NLSN-PM) at the 47th, 48th and 49th positions of the NLS (x63). (N=3) **(E)** WT and the phospho-mimetic (YFP-SPANX-APM) and dephospho-mimetic mutants (YFP-SPANX-AN-PM) of the 5th, 13th , 18th, 28th, 47th, 48th and 49th positions of the WT sequence (x40 and x63). The zoomed section appears framed by a dotted line Scale bar: 15 µm. (N=3). **(F)** Graph showing the CTCF intensity/YFP positive cells of the YFP-SPANX-APM vs YFP-SPANX-AN-PM mutants. The data shown in the graph correspond to the mean of three independent experiments, and error bars indicate the SEM. *P<0.05 (Students T-test).

# Supplementary Figure 2

**Supplementary Figure 2.** 3D reconstruction of the localization YFP-SPANX-A and YFP-SPANX-B isoforms in HEK293T cells. Confocal microscopy images showing representative examples of HEK293T cells transfected with expression plasmids enconding **A)** YFP-SPANX-A and **B)** YFP-SPANX-B.

# Supplementary Figure 3


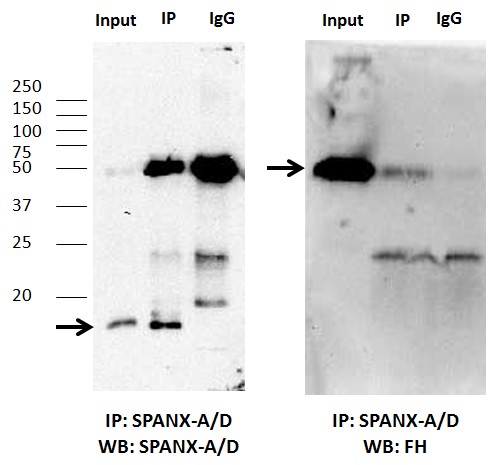


**Supplementary Figure 3. Study of the interaction between SPANX-A/D and FH in human spermatozoa by Western Blotting.** Immunoprecipitation of SPANX-A/D in human spermatozoa (left panel). Co immunoprecipitation of SPANX-A/D and FH (right panel). Negative controls were performed with non-specific IgGs for immunoprecipitation.(N=3).
